# Supplementary figures and images for: Optimizing colormaps with consideration for color vision deficiency to enable accurate interpretation of scientific data
Source: PLoS One. 2018 Aug 1;13(7):e0199239. doi: 10.1371/journal.pone.0199239 (PMC6070163; doi:10.1371/journal.pone.0199239)

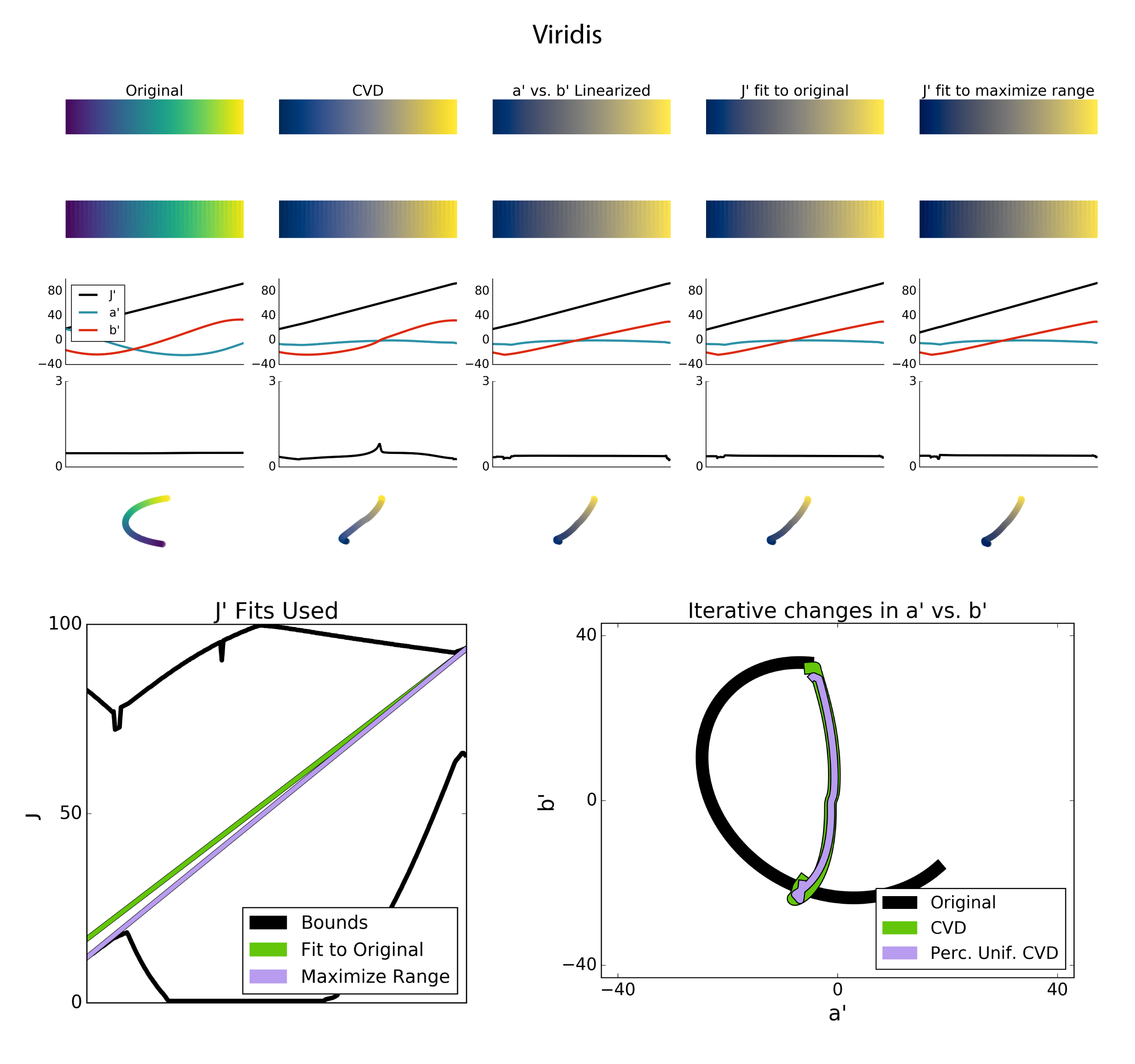

Supplement: S1 File — Zip file of example optimization figures for multiple colormaps we tested. This includes the iteration image (colormap conversion to CVD, interpolation of a′ vs. b′, and the two J′ linearization methods), a′ vs. b′ change overlay, and a comparison of the two fits used for J′. (ZIP) [file pone.0199239.s001.zip › viridis.png]

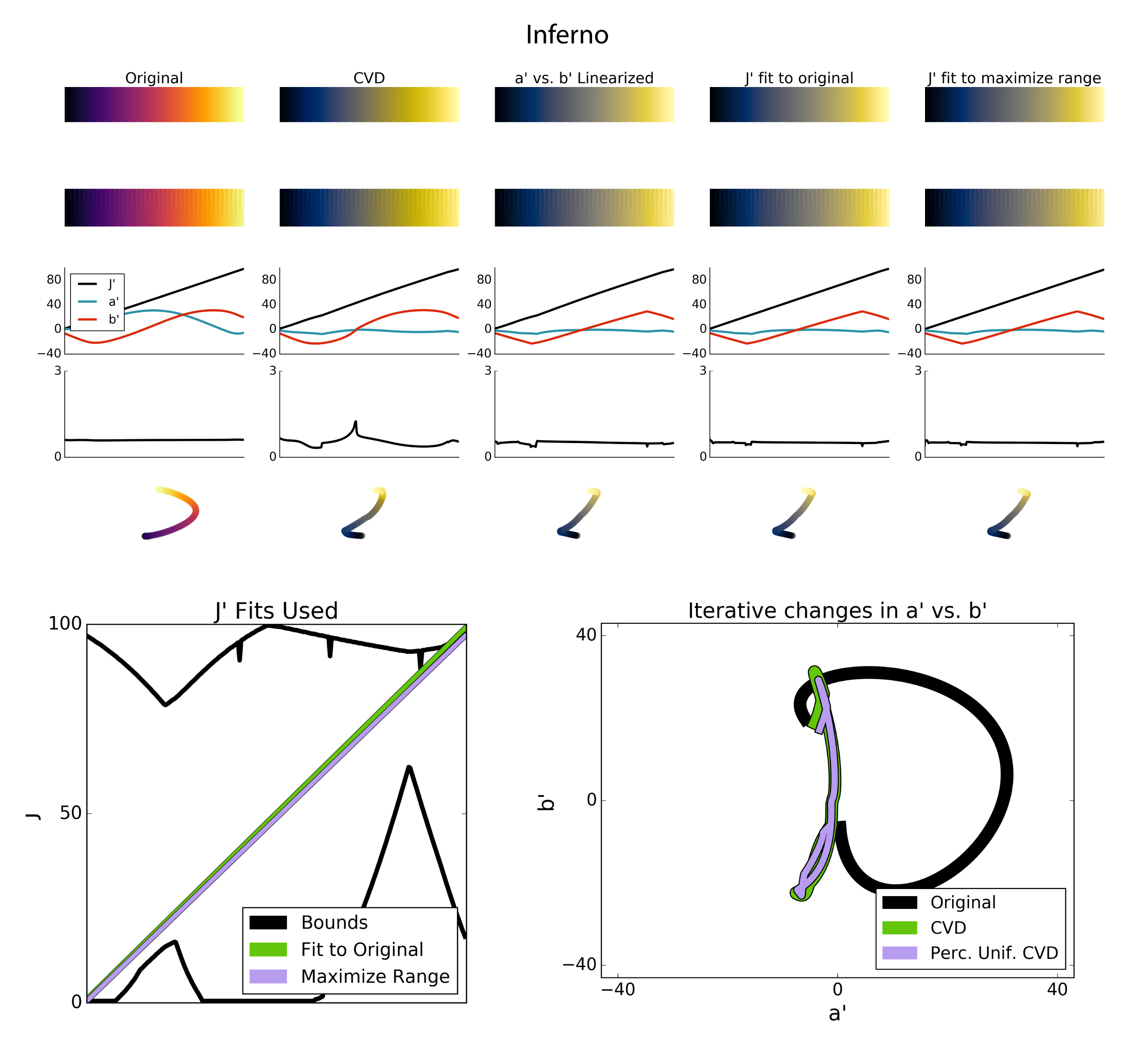

Supplement: S1 File — Zip file of example optimization figures for multiple colormaps we tested. This includes the iteration image (colormap conversion to CVD, interpolation of a′ vs. b′, and the two J′ linearization methods), a′ vs. b′ change overlay, and a comparison of the two fits used for J′. (ZIP) [file pone.0199239.s001.zip › inferno.png]

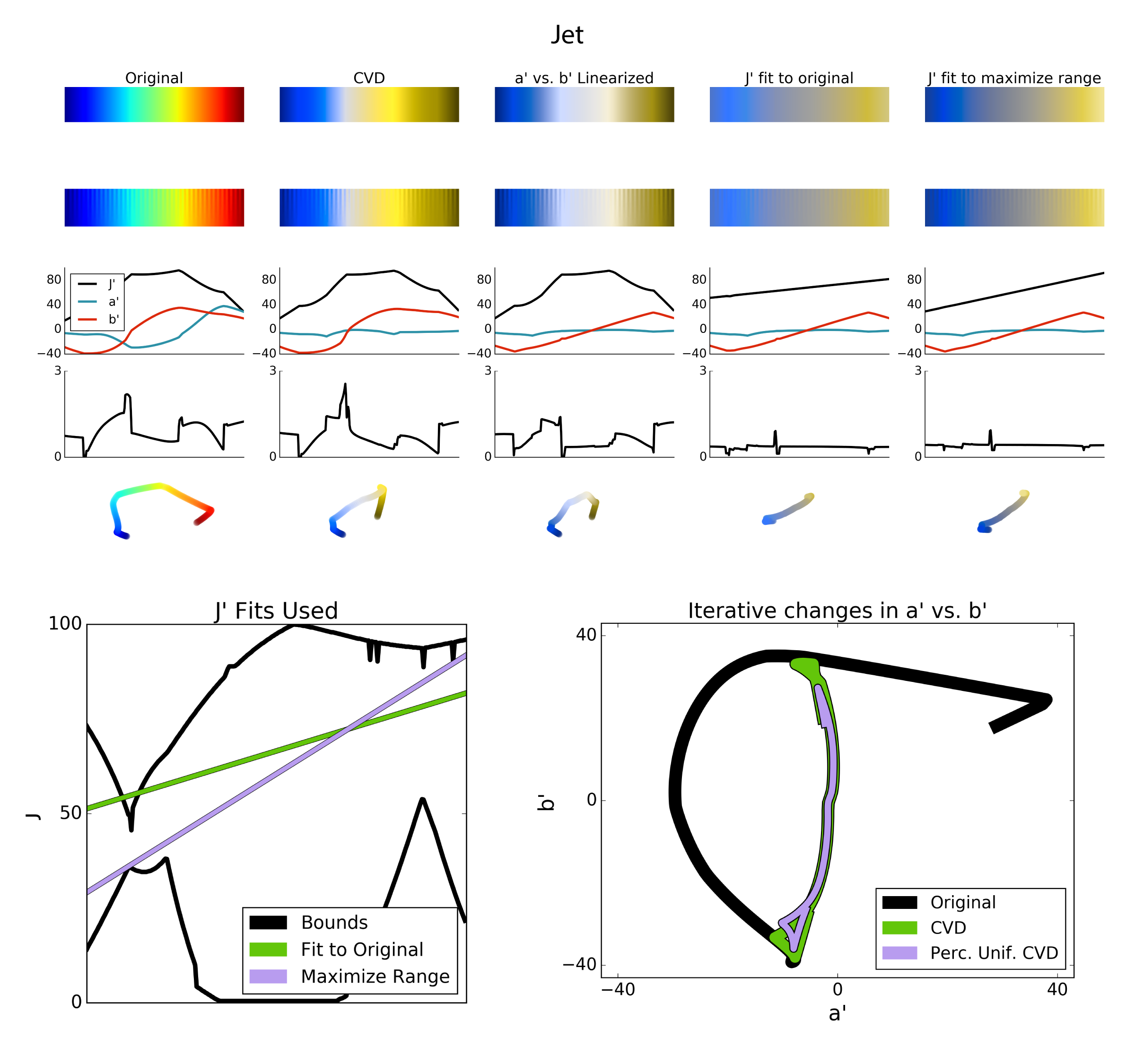

Supplement: S1 File — Zip file of example optimization figures for multiple colormaps we tested. This includes the iteration image (colormap conversion to CVD, interpolation of a′ vs. b′, and the two J′ linearization methods), a′ vs. b′ change overlay, and a comparison of the two fits used for J′. (ZIP) [file pone.0199239.s001.zip › jet.png]

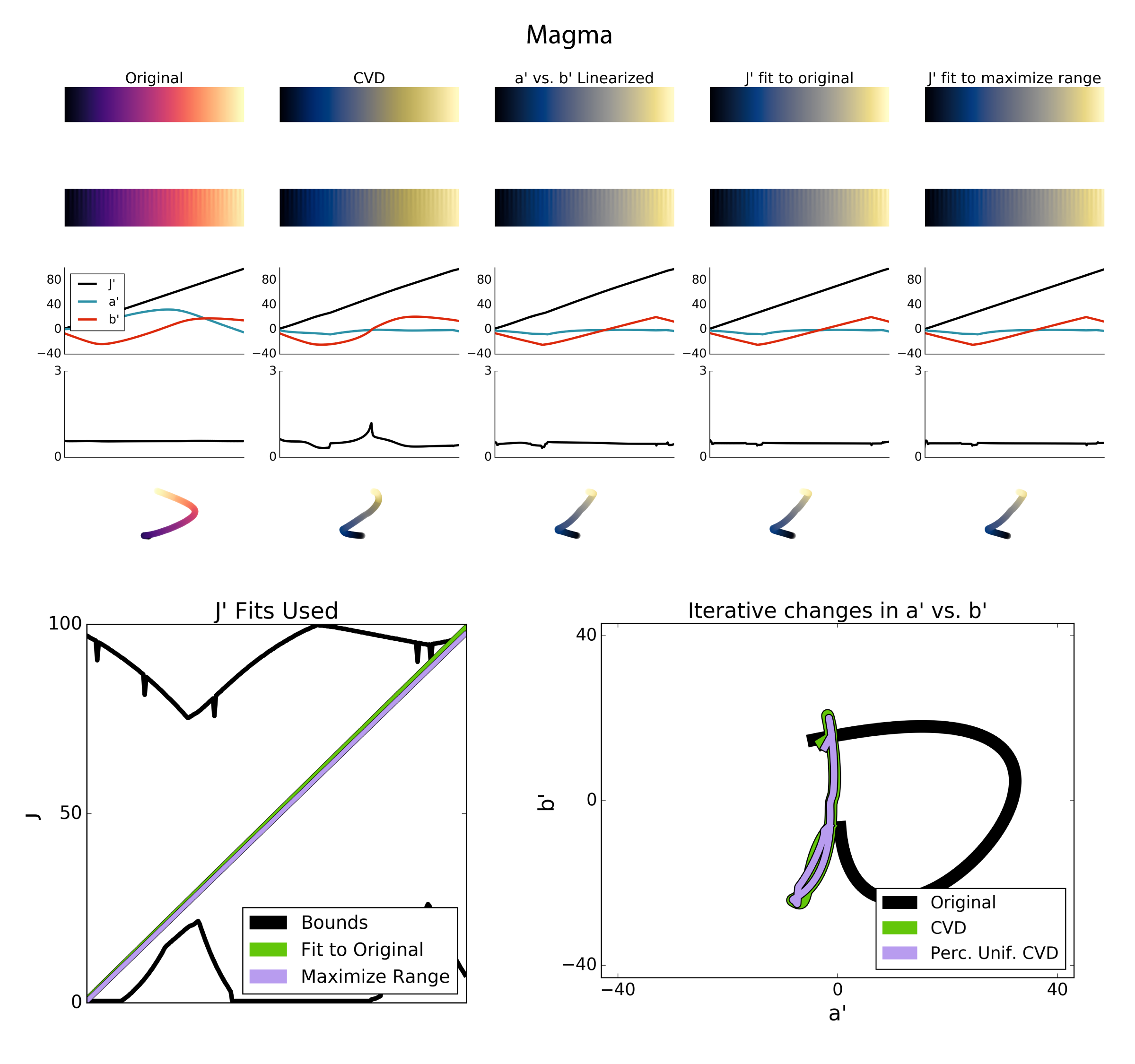

Supplement: S1 File — Zip file of example optimization figures for multiple colormaps we tested. This includes the iteration image (colormap conversion to CVD, interpolation of a′ vs. b′, and the two J′ linearization methods), a′ vs. b′ change overlay, and a comparison of the two fits used for J′. (ZIP) [file pone.0199239.s001.zip › magma.png]

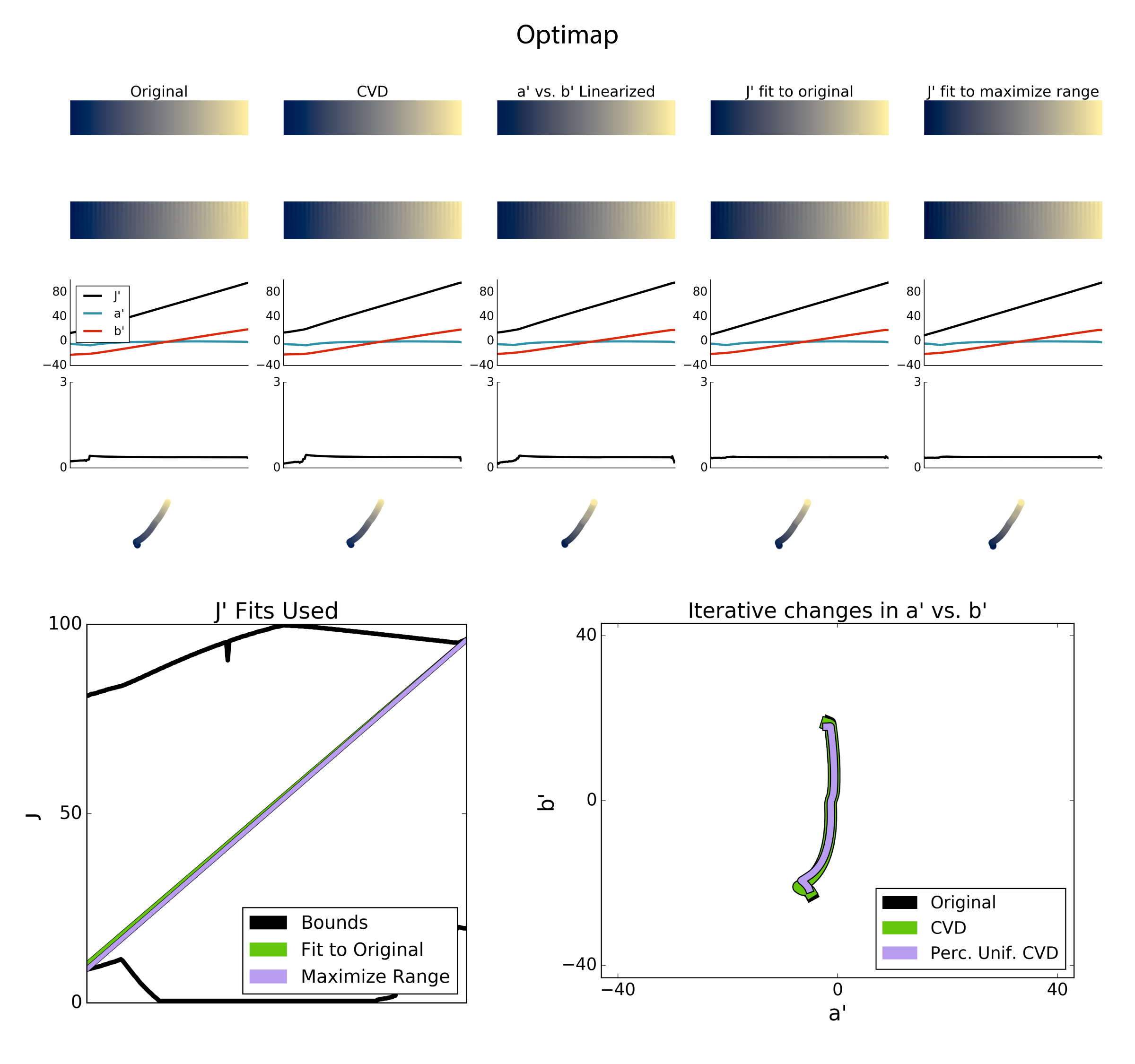

Supplement: S1 File — Zip file of example optimization figures for multiple colormaps we tested. This includes the iteration image (colormap conversion to CVD, interpolation of a′ vs. b′, and the two J′ linearization methods), a′ vs. b′ change overlay, and a comparison of the two fits used for J′. (ZIP) [file pone.0199239.s001.zip › optimap.png]

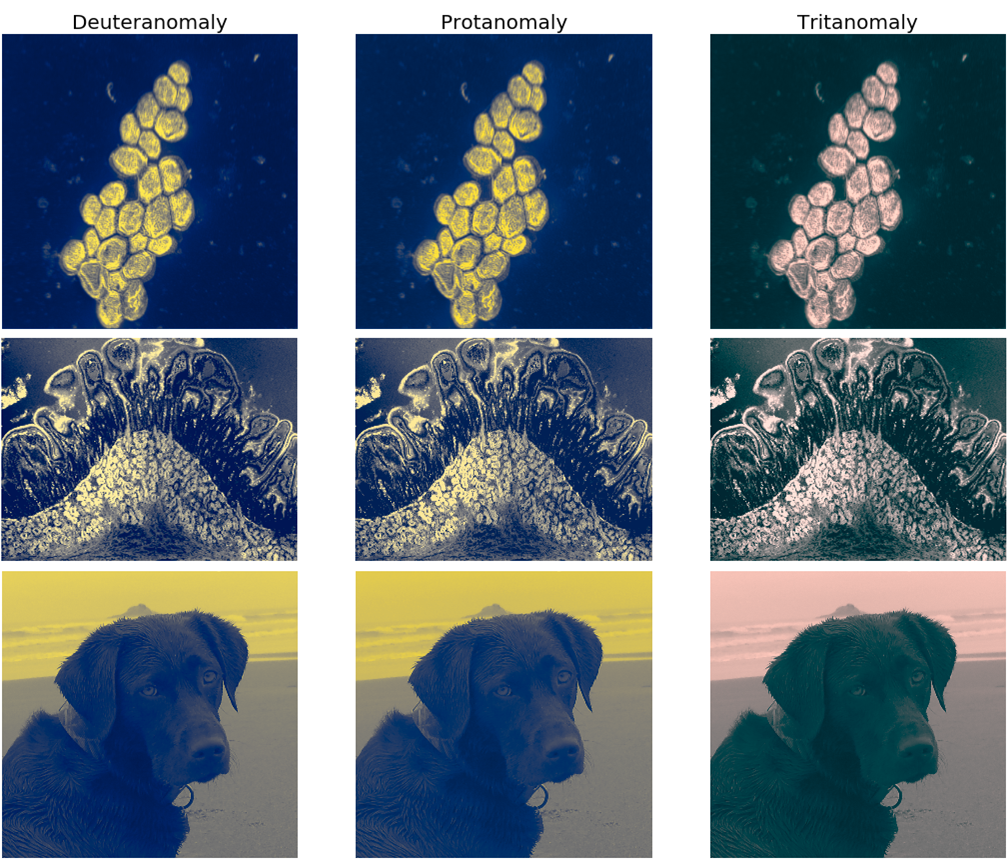

Supplement: S4 File — Three images overlaid with the cividis colormap as it would appear with one of the three forms of CVD (deuteranomaly, protanomaly, and tritanomaly), severity 100. Middle image,"4x autofluor.tif", collected from imagej.nih.gov/ij/docs/examples/IJ-M&M08-Figures.zip. (PNG) [file pone.0199239.s004.png]
